# *Plasmodium falciparum* CRK4 links early mitotic events to the onset of S-phase during schizogony

**DOI:** 10.1128/mbio.00779-23

**Published:** 2023-06-22

**Authors:** Marta Machado, Severina Klaus, Darius Klaschka, Julien Guizetti, Markus Ganter

**Affiliations:** 1 Center for Infectious Diseases, Heidelberg University Hospital, Heidelberg, Germany; 2 Graduate Program in Areas of Basic and Applied Biology, Instituto de Ciências Biomédicas Abel Salazar, Universidade do Porto, Porto, Portugal; UT Southwestern Medical Center, Dallas, Texas, USA

**Keywords:** malaria, cell cycle, *Plasmodium falciparum*, kinase, microtubules, DNA replication

## Abstract

*Plasmodium falciparum* proliferates through schizogony in the clinically relevant blood stage of infection. During schizogony, consecutive rounds of DNA replication and nuclear division give rise to multinucleated stages before cellularization occurs. Although these nuclei reside in a shared cytoplasm, DNA replication and nuclear division occur asynchronously. Here, by mapping the proteomic context of the S-phase-promoting kinase *Pf*CRK4, we show that it has a dual role for nuclear-cycle progression: *Pf*CRK4 orchestrates not only DNA replication, but in parallel also the rearrangement of intranuclear microtubules from hemispindles into early mitotic spindles. Live-cell imaging of a reporter parasite showed that these microtubule rearrangements coincide with the onset of DNA replication. Together, our data render *Pf*CRK4 a key factor for nuclear-cycle progression, linking entry into S-phase with the initiation of mitotic events. In part, such links may compensate for the absence of canonical cell cycle checkpoints in *P. falciparum*.

**IMPORTANCE:**

The human malaria parasite *Plasmodium falciparum* proliferates in erythrocytes through schizogony, forming multinucleated stages before cellularization occurs. In marked contrast to the pattern of proliferation seen in most model organisms, *P. falciparum* nuclei multiply asynchronously despite residing in a shared cytoplasm. This divergent mode of replication is, thus, a good target for therapeutic interventions. To exploit this potential, we investigated a key regulator of the parasite’s unusual cell cycle, the kinase *Pf*CRK4 and found that this kinase regulated not only DNA replication but also in parallel the rearrangement of nuclear microtubules into early mitotic spindles. Since canonical cell cycle checkpoints have not been described in *P. falciparum* parasites, linking entry into S-phase and the initiation of mitotic events via a kinase, may be an alternative means to exert control, which is typically achieved by checkpoints.

## INTRODUCTION

*Plasmodium falciparum* is the etiologic agent of the most severe form of human malaria, which remains a major cause of global morbidity and mortality (1). In the clinically relevant blood stage of infection, this unicellular eukaryotic parasite proliferates through a divergent cell cycle mode called schizogony, where alternating rounds of S-phase and nuclear division form multinucleated cells before cellularization occurs (2 - 4). In most multinucleated cells, for example, the marine eukaryote *Sphaeroforma arctica* or the early *Drosophila* embryo (5, 6), nuclei multiply synchronously. Yet, asynchronous nuclear multiplication can also be seen in the filamentous fungus *Ashbya gossypii* (7). Here, asynchrony arises from nucleus-intrinsic mechanisms, like variations in transcription, and restricted diffusion of cytoplasmic factors as nuclei establish their own cytoplasmic territories, which insulate their division cycle (8 - 10). *P. falciparum* nuclei also undergo asynchronous DNA replication and nuclear division despite sharing a common cytoplasm (3, 11 - 13). In contrast to *A. gossypii*, where nuclei are regularly spaced approximately 5 µm apart (9), *P. falciparum* nuclei are in much closer proximity, sometimes less than 100 nm (3). This suggests a divergent and locally restricted regulation of nuclear cycle progression (14 - 16).

Typically, cyclins and cyclin-dependent kinases (CDKs) drive the timely progression through the cell cycle (17, 18). Although no canonical G1, S- and M-phase cyclins could be identified (19, 20), the *P. falciparum* genome harbors CDK-like kinases and several related kinases, such as the cdc2-related kinase 4 (*Pf*CRK4) (21). Conditional depletion of *Pf*CRK4 suppressed DNA replication in the blood stage of infection and arrested parasites have large hemispindle-like microtubule structures (12, 22), indicating that *Pf*CRK4 is not required for hemispindle formation. Phosphoproteomic profiling of *Pf*CRK4 identified a set of potential effector proteins, which are likely involved in origin of replication firing (22). This suggested that *Pf*CRK4 is a major S-phase promoting factor in *P. falciparum*. However, a detailed understanding of the regulatory circuits that drive asynchronous nuclear cycles during blood-stage schizogony is still missing.

To gain a better understanding of *Pf*CRK4’s role for asynchronous nuclear cycles, we profiled the proteomic context of this kinase and found several microtubule-associated proteins in the vicinity of *Pf*CRK4, suggesting an additional molecular function besides regulating DNA replication. Further supporting this notion, we detected *Pf*CRK4 predominantly at intranuclear microtubule foci. Super-resolution live-cell imaging showed that major microtubule rearrangements depend on *Pf*CRK4 and coincide with the onset of DNA replication. Our data integrate the dynamics of microtubule structures and DNA replication, which have been independently used to assess nuclear-cycle progression in *P. falciparum*. These data also expand the repertoire of molecular functions assigned to *Pf*CRK4, rendering it a key regulator of nuclear-cycle progression during asynchronous nuclear cycles.

## RESULTS

### Profiling the *Pf*CRK4 proximity proteome

**Fig 1:**
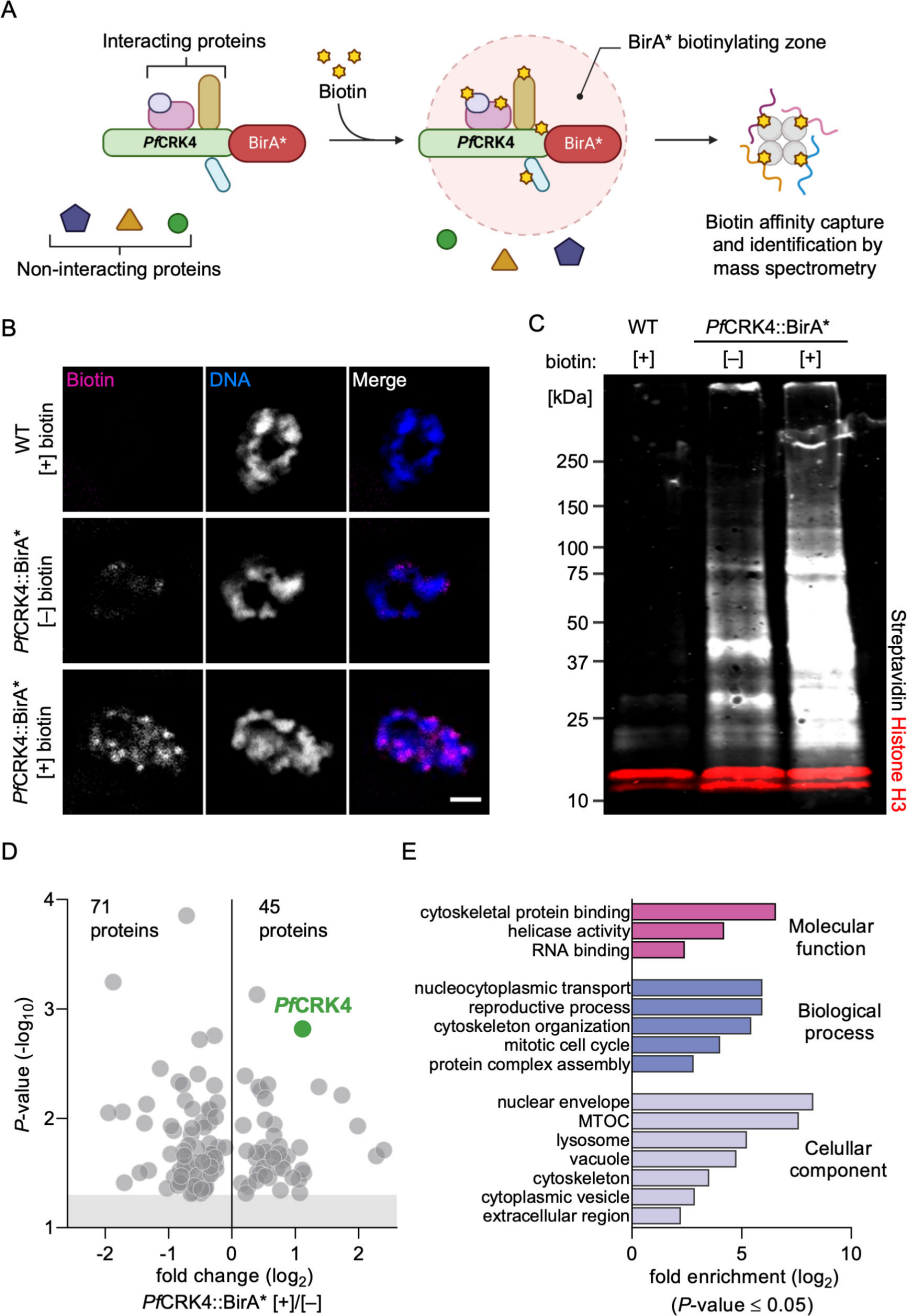
Profiling the proximity proteome of *Pf*CRK4 identified a potential link to microtubules. (A) Scheme illustrating the principal steps of proximity-based labeling via BioID, created with BioRender.com. (B) Biotinylated proteins predominantly localize to a small region of *P. falciparum* nuclei; scale bar, 2 µm. (C) Western blot analysis showed specific biotinylation in *Pf*CRK4::BirA* parasites. IRDye 800CW streptavidin was used to detect biotinylated proteins (grey), α-histone H3 was used as a loading control and visualized by IRDye 680CW goat anti-rabbit IgG (red). (D) Underrepresented and overrepresented proteins in the vicinity of *Pf*CRK4::BirA* parasites [+] 50 µM biotin relative to [–] biotin. Shown are mean values of triplicates; grey shaded region indicates a *P*-value ≥0.05 (Student’s *t*-test, two-tailed, equal variance). (E) Gene ontology term enrichment analysis of 44 proteins (excluding *Pf*CRK4) that are overrepresented in the vicinity of *Pf*CRK4, calculated using the built-in tool at PlasmoDB.org.

To analyze the role of the S-phase promoting kinase *Pf*CRK4 for nuclear-cycle progression, we mapped its proteomic context. For this, we endogenously fused *Pf*CRK4 with the promiscuous biotin ligase BirA*, which allows the biotinylation of proteins within a radius of ~20 nm (23) (Fig. 1A; Fig. S1A and B). To functionally characterize the *Pf*CRK4::BirA* expressing line, ring-stage parasites were grown in the presence or absence of additional 50 µM biotin for 24 h and subsequently stained with fluorescent streptavidin to detect biotinylated proteins (Fig. 1B). Wild-type (WT) parasites cultured with additional biotin showed no detectable signal. In *Pf*CRK4::BirA* parasites, the approximately 0.8 µM biotin of the standard culture medium was sufficient for a detectable biotin labeling, as previously reported (24, 25). The signal strongly increased, when we supplemented the culture media with 50 µM biotin and we found biotinylated proteins predominantly localized to the nucleus of *Pf*CRK4::BirA* parasites (Fig. 1B; Fig. S1C). This is in agreement with the spatiotemporal expression of *Pf*CRK4 fused to GFP (Fig. S1A, B, D) and the previously reported localization of *Pf*CRK4 (22). Western blot analysis further supported these findings (Fig. 1C), indicating that the *Pf*CRK4::BirA* fusion protein is active.

To profile the proteomic context of *Pf*CRK4, we enriched for biotinylated proteins from *Pf*CRK4::BirA* [+] and [–] 50 µM biotin as well as WT parasites [+] 50 µM biotin and analyzed triplicate samples by quantitative label-free mass spectrometry. Due to low reproducibility between triplicates (Fig. S2A), we refrained from a quantitative analysis of WT [+] 50 µM biotin samples, which were intended to serve as negative control. Next, we asked which proteins were enriched in the proximity of *Pf*CRK4::BirA* by comparing the abundance of proteins in [+] over [–] biotin samples (Fig. 1D; Table S1A). We found 44 proteins to be significantly overrepresented in the proximity of *Pf*CRK4, including the bait protein, and 71 proteins significantly underrepresented. The set of overrepresented proteins included four proteins (PF3D7_0624600, PF3D7_0209800, PF3D7_1357400, and PF3D7_0303500) that were also less phosphorylated in the absence of *Pf*CRK4 (Fig. S2B; Table S1A) (22). To further validate our proximity proteome data, we assessed the localization of one of these candidates, the putative spindle pole body protein (*Pf*SPB, PF3D7_0303500) (Fig. S2C through E). We detected *Pf*SPB at the parasite’s centrosome, also called centriolar plaque, which organizes intranuclear microtubules (12, 26, 27). At the centriolar plaque, *Pf*SPB localized between the extranuclear centrin and the intranuclear tubulin (Fig. S2E). This localization is consistent with the bulk labeling of biotinylated proteins and the localization of *Pf*CRK4::GFP (Fig. 1B; Fig. S1C and D).

Next, we used gene ontology (GO) term enrichment analysis to examine the functional characteristics of the underrepresented and overrepresented sets of proteins in the proximity of *Pf*CRK4. While underrepresented proteins appear mainly involved in translation (Fig. S3A), the enriched GO terms of overrepresented proteins suggested an association of *Pf*CRK4 with the cytoskeleton (Fig. 1E). STRING analysis (28) of the overrepresented proteins clustered *Pf*CRK4 with β-tubulin, suggesting a link between *Pf*CRK4 and microtubule structures of the cytoskeleton (Fig. S3B). Together with the previous observation that *Pf*CRK4-depleted parasites show prominent intranuclear microtubule structures (22), these data prompted us to investigate a potential additional role for *Pf*CRK4 besides directing DNA replication.

### *Pf*CRK4 associates with nuclear microtubules

**Fig 2:**
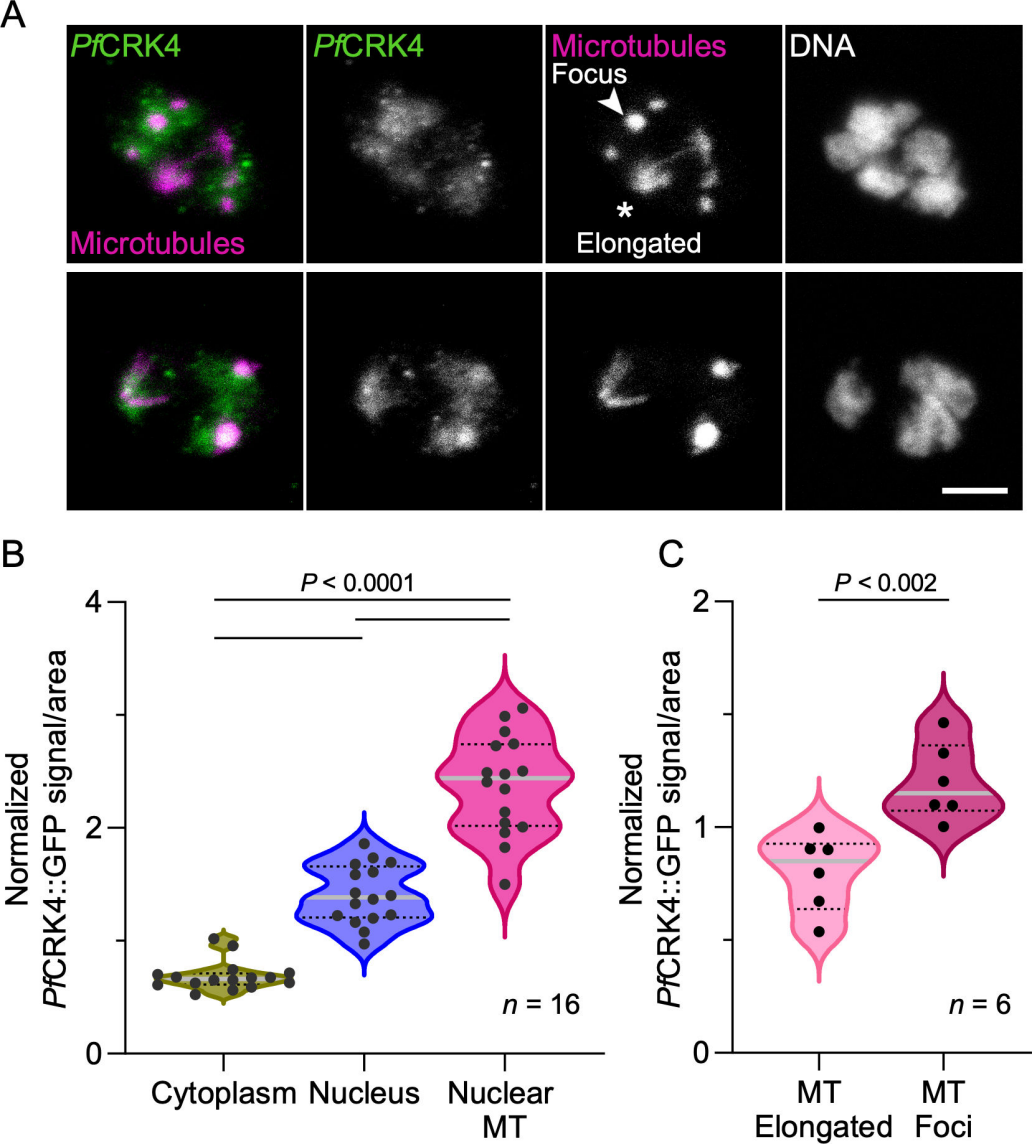
*Pf*CRK4 localized predominantly to focal microtubule structures in the nucleus. (A) Immunofluorescence showing uneven distribution of *Pf*CRK4 relative to nuclear microtubule structures in a multinucleated parasite; scale bar, 2 µm. (B) *Pf*CRK4 predominantly localizes to nuclear microtubule (MT) structures; *P*-value, one-way ANOVA with Tukey’s multiple comparison posttest. Signals were normalized to the total fluorescence of the whole cell. (C) *Pf*CRK4 associates more with focal than with elongated microtubule structures; *P*-value, unpaired Student’s *t*-test with Welch’s correction. Signals were normalized to the total fluorescence of nuclear microtubules. Data shown in B, C are representative data from two independent experiments, quantifications were done on images from one replicate; solid grey lines, median; dashed black lines, interquartile range.

Microtubules display a dynamic rearrangement during the nuclear cycle (12, 29). Initially, microtubules form a so-called hemispindle, consisting of relatively long microtubules that radiate from the centriolar plaque into the nucleoplasm. Later they rearrange into short microtubules, which build the early mitotic spindle. The mature mitotic spindle eventually extends to partition the duplicated genome. After nuclear division, hemispindles re-appear in sister nuclei as the remnants of the divided anaphase spindle (12, 29). To investigate the hypothesis that *Pf*CRK4 contributes to microtubule dynamics, we quantified its subcellular localization (Fig. 2; Fig. S4 and S5). We detected *Pf*CRK4 predominantly in the nucleus and within the nuclei, the highest *Pf*CRK4 signal per area was observed at microtubule structures (Fig. 2A and B; Fig. S4). Comparing elongated with focal nuclear microtubule structures, we found *Pf*CRK4 predominantly associated with microtubule foci (Fig. 2A and C; Fig. S4). Together, these data indicated a link between *Pf*CRK4 and intranuclear microtubule structures.

### Rescued *Pf*CRK4 function triggers DNA replication and rearrangement of nuclear microtubules

**Fig 3:**
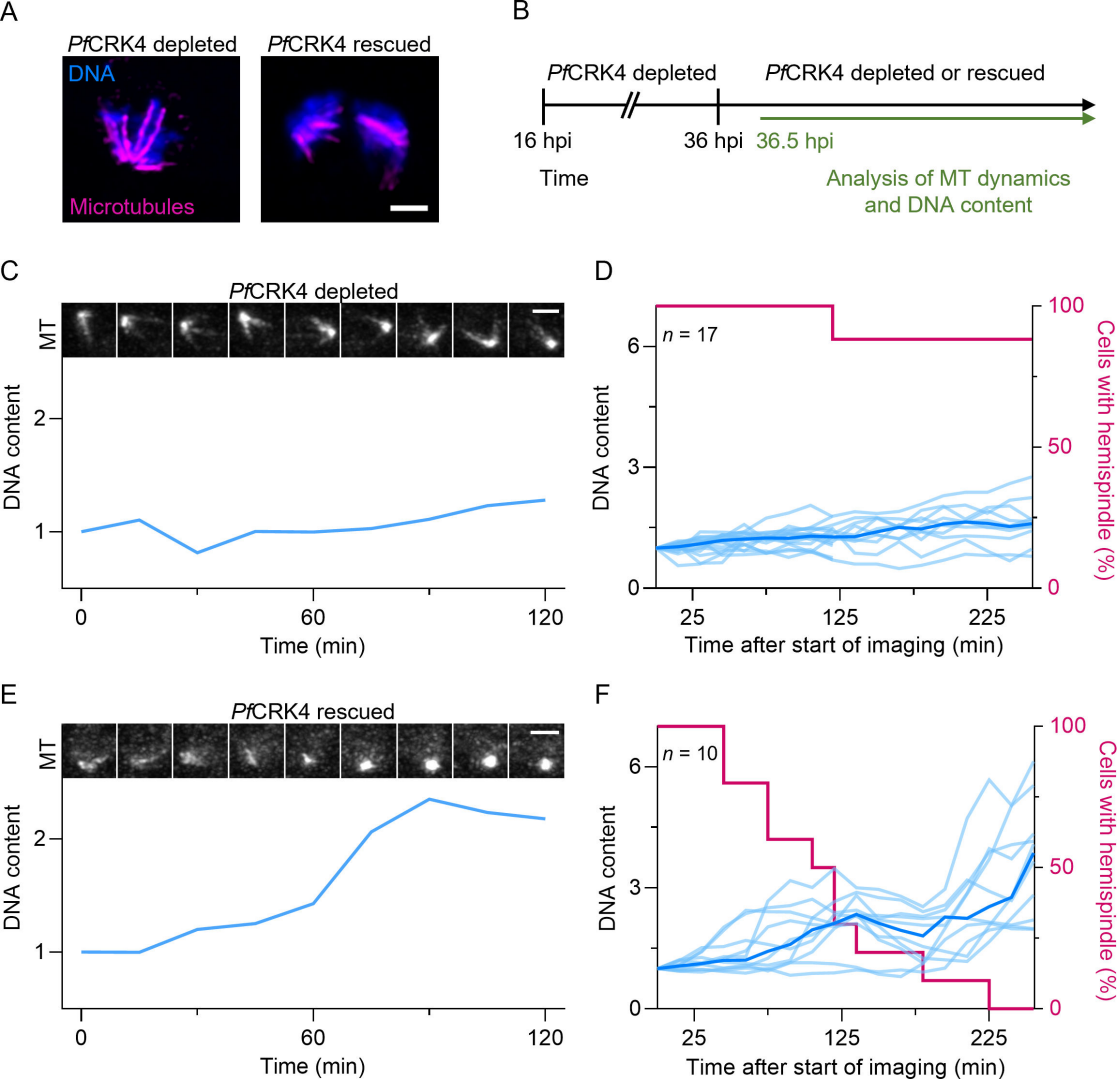
Rescued *Pf*CRK4 function triggers rearrangement of nuclear microtubules. (A) Immunofluorescence of nuclear microtubules in *Pf*CRK4-depleted and *Pf*CRK4-active parasites; scale bar, 1 µm. (B) Scheme of the timing of rescued *Pf*CRK4 function and subsequent super-resolution live-cell imaging; hpi, hours post invasion. (C) Representative images of microtubule (MT) structures (top) and normalized DNA content (bottom) over time in a *Pf*CRK4-depleted parasite, single z-slice, scale bar, 2 µm. (D) Quantification of hemispindle structures and the DNA content of *Pf*CRK4-depleted parasites; light blue, single cell signals; dark blue, mean signal. (E) Representative images of microtubule (MT) structures (top) and normalized DNA content (bottom) over time in a parasite with rescued *Pf*CRK4 function, single z-slice, scale bar, 2 µm. (F) Quantification of hemispindle structures and the DNA content of parasites with rescued *Pf*CRK4 function; light blue, single cell signals; dark blue, mean signal. C–F, DNA content was normalized to the total fluorescence of the first time point (36.5 hpi). Data shown in D and F are representative data from two independent imaging sessions.

To further explore this potential link, we employed the previously established *Pf*CRK4::HA::DD parasite, which permits the inducible depletion of *Pf*CRK4 by the removal of the small molecule Shield-1 (22). First, we confirmed previous data that *Pf*CRK4-depleted parasites display prominent intranuclear microtubule structures that resemble hemispindles (Fig. 3A) (22). Next, we analyzed the effect of rescued *Pf*CRK4 function on these microtubule structures. For this, we depleted *Pf*CRK4 until approximately 36 h post-invasion, when the *Pf*CRK4 phenotype is well established (22). Then, we either kept the kinase depleted or we rescued *Pf*CRK4 function by addition of Shield-1 (Fig. 3B). Subsequently, we analyzed the dynamics of intranuclear microtubules and the nuclear DNA content. For this, we stained the parasites with the live-cell compatible dyes SPY555-tubulin and 5′-SiR-Hoechst and imaged single cells at super-resolution for 4 h.

In *Pf*CRK4-depleted cells, we found prominent intranuclear microtubule structures, which consisted of constantly polymerizing and depolymerizing microtubules and a profoundly suppressed DNA replication (Fig. 3C and D; movie S1). In contrast, when *Pf*CRK4 function was rescued, the prominent hemispindle-like structures rearranged into focal microtubule structures and the DNA content of these nuclei increased (Fig. 3E and F; movie S2), further coupling intranuclear microtubule dynamics to *Pf*CRK4. Previous data have shown that inhibitors of microtubule dynamics do not affect DNA replication in *Plasmodium* spp. (30 - 32). This suggests that orderly microtubule rearrangements are not a prerequisite for DNA replication. To test this, we treated *Pf*CRK4-depleted cells with the microtubule-stabilizing compound paclitaxel (33) and then rescued *Pf*CRK4 function (Fig. S6). Next, we analyzed the microtubule structures by immunofluorescence (Fig. S6B) and quantified the DNA content by flow cytometry (Fig. S6C) (22, 34, 35). Although we readily detected large aberrant microtubule structures in these cells, DNA replication commenced when *Pf*CRK4 was active (Fig. S6), confirming that accurate microtubule rearrangement is not necessary for DNA replication to commence.

### Nuclear microtubule rearrangement coincides with the onset of S-phase and is independent of processive DNA replication

**Fig 4:**
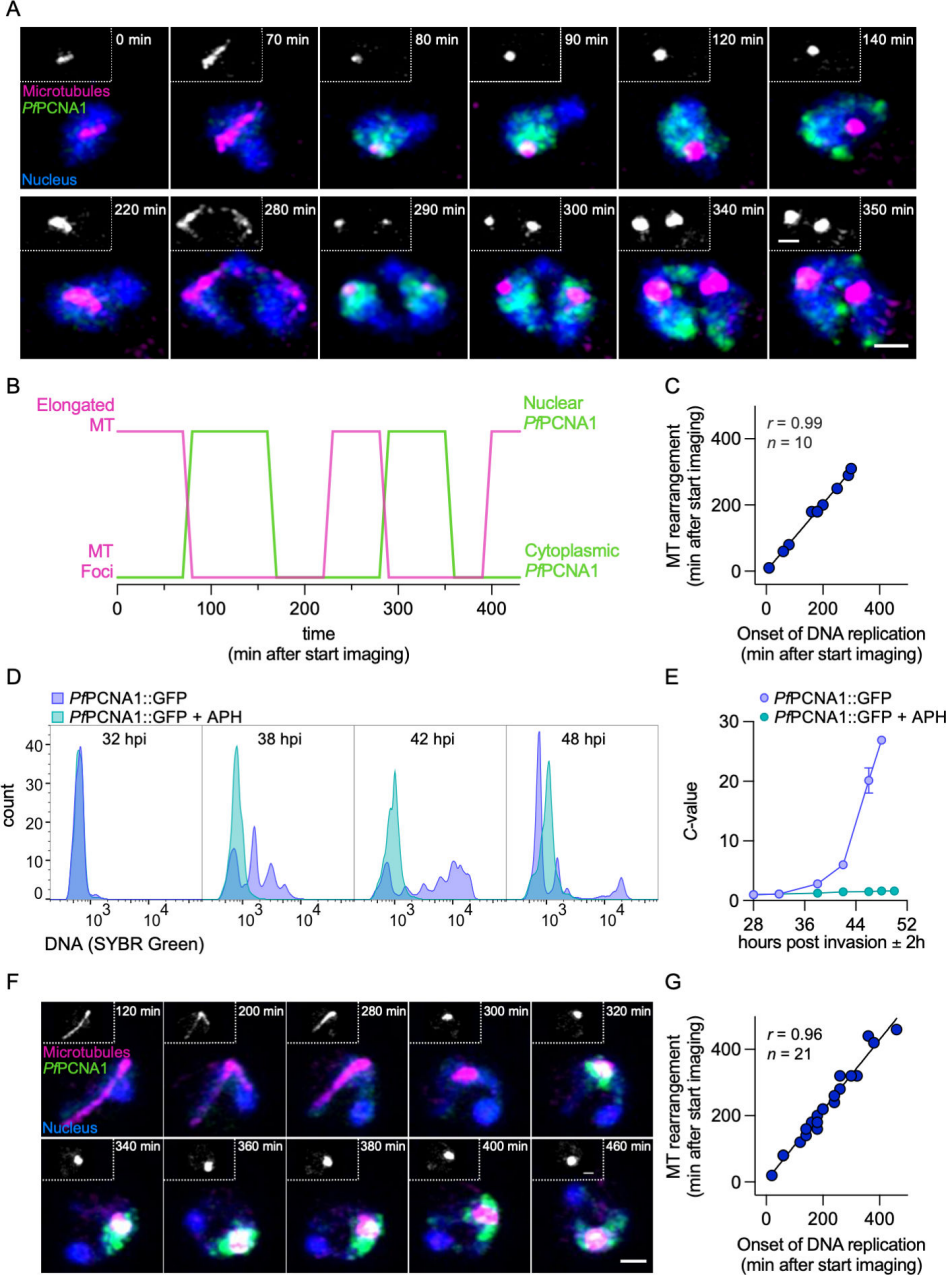
Rearrangement of nuclear microtubules coincides with the onset of S-phase and is independent of processive DNA replication. (A) Super-resolution live-cell imaging of microtubule structures (SPY650-magenta) in a nuclear cycle sensor line, where nuclei are marked with mCherry (blue) and nuclear accumulation of *Pf*PCNA1::GFP (green) marks DNA replication; inserts, microtubule signal; scale bars, 1 µm. (B) Timing of elongated and focal nuclear microtubule structures (MT) relative to the localization of *Pf*PCNA1::GFP, with nuclear *Pf*PCNA1::GFP marking DNA replication of the parasite shown in A. (C) Correlation of the timing of microtubule rearrangement and the onset of DNA replication. Data from two independent imaging sessions. (D) Representative flow cytometry histograms showing the DNA content (SYBR Green I) over time of parasites treated with 15 µM of APH from 28 hpi onwards. (E) DNA content of *Pf*PCNA1::GFP parasites treated with APH or solvent; mean ± SD of triplicates (representative of two biological replicates); ring-stage DNA content defined as 1 *C*. For the 46 h and 48 h time points, only the late schizont population was gated. (F) Super-resolution live-cell imaging of *Pf*PCNA1::GFP parasites treated with 15 µM APH from 28 hpi onwards and imaged starting at 32 hpi; scale bars, 1 µm. (G) Correlation of the timing of microtubule rearrangement and the onset of DNA replication in APH treated *Pf*PCNA1::GFP parasites. Data from one imaging session.

As depletion of *Pf*CRK4 arrests normal parasite development, we next investigated the temporal coordination of intranuclear microtubule rearrangement and DNA replication without perturbation. For this, we stained microtubules with SPY650-tubulin in a *P. falciparum* nuclear cycle sensor line (3). This line expresses the red-fluorescent protein mCherry in all nuclei and the proliferating cell nuclear antigen 1 (*Pf*PCNA1) fused to GFP. *Pf*PCNA1::GFP transiently accumulates only in those nuclei that replicate their DNA, and hence, DNA replication and nuclear division events can be tracked. Using super-resolution live-cell microscopy, we imaged cells with a single nucleus and found that hemispindles with dynamic microtubules formed well before *Pf*PCNA1::GFP accumulated in the nuclei, which marks the onset of DNA replication. Thus, the presence of a hemispindle is currently the first visible cue of an imminent S-phase. Coinciding with the start of DNA replication, hemispindles rearranged into short microtubule structures that likely localized close to the centriolar plaque (Fig. 4A through C; movie S3). Once the subsequent nuclear division concluded, hemispindle structures reappeared in sister nuclei. Again, the rearrangement of these second-generation hemispindles coincided with the onset of DNA replication in those nuclei (Fig. 4A through C; movie S3), suggesting a tight temporal coordination of these two events throughout schizogony. We also found that *Pf*PCNA1::GFP initially accumulated adjacent to the focal microtubule signal at the centriolar plaque and over time translocated to distal regions of the nucleus (Fig. 4A; movie S3), suggesting that DNA close to the early mitotic spindle is replicated before DNA in the nuclear periphery, such as the telomeres with *var* genes (36). Throughout S-phase and after it concluded, the focal microtubule signals appeared to increase (Fig. 4A inset; movie S3), potentially reflecting the maturation of the mitotic spindle.

Next, we tested if microtubule rearrangements are a consequence of processive DNA replication or whether both processes are directed by *Pf*CRK4 in parallel. To distinguish these scenarios, we used the DNA polymerase inhibitor aphidicolin (APH) (37), which profoundly suppressed DNA replication at a concentration of 15 µM in the *P. falciparum* nuclear cycle sensor line (Fig. 4D and E). In the presence of APH, microtubules rearranged at the onset of S-phase as marked by the nuclear accumulation of *Pf*PCNA1::GFP, similar to untreated cells (Fig. 4A, C, F and G). This indicates that *Pf*CRK4 directs DNA replication and intranuclear microtubule rearrangement in parallel. In contrast to untreated cells, *Pf*PCNA1 remained in the nucleus over extended amounts of time (3), further supporting the inhibition of DNA replication by APH (Fig. 4F).

**Fig 5:**
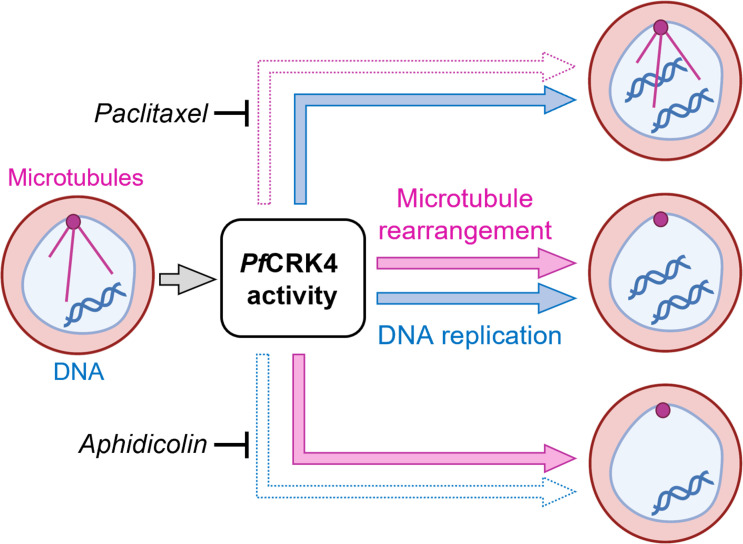
*Pf*CRK4 is a critical regulator of nuclear-cycle progression. Working model summarizing the molecular functions of *Pf*CRK4, which in parallel directs microtubule rearrangements and DNA replication at the onset of S-phase.

Together, our data determined the temporal coordination of nuclear microtubule dynamics and DNA replication and found that not only DNA replication but also hemispindle-to-mitotic spindle rearrangement depend on *Pf*CRK4, rendering this kinase a master regulator of nuclear-cycle progression (Fig. 5).

## DISCUSSION

In the clinically relevant blood stage of infection, *P. falciparum* proliferates through schizogony, during which asynchronous nuclear cycles occur in a multinucleated cell. The parasite’s unusual mode of replication is likely a good target for therapeutic interventions. Yet, to exploit this potential, insights into the underlying molecular mechanisms are needed and we, therefore, further analyzed the role of the S-phase promoting kinase *Pf*CRK4 (22). Combining proteomic and advanced imaging approaches, we provide evidence that intranuclear microtubules rearrange from hemispindle into early mitotic spindles at the onset of DNA replication, and that both processes are linked via *Pf*CRK4.

During normal nuclear-cycle progression, hemispindles appear well before S-phase commences (Fig. 4A and B; movie S3). Thus, in contrast to what has been proposed (38), the large hemispindle-like structures seen in *Pf*CRK4-depleted parasites are consistent with a developmental arrest just prior to S-phase, rather than representing a *Pf*CRK4-depletion phenotype during nuclear division (Fig. 3 and 4). Whether the hemispindle has a biological function or whether it is a result of local high concentrations of tubulin that self-assemble into microtubules, remains unclear (12). Similarly, the events that occur after the microtubule rearrange and that eventually lead to the attachment of the duplicated chromosomes to the mature mitotic spindle are not understood.

The molecular details of how *Pf*CRK4 regulates the rearrangement of microtubules at the onset of S-phase are unknown. Mammalian CDK1 can phosphorylate β-tubulin and phosphorylated β-tubulin incorporates only poorly into microtubules (39). Yet, in *Pf*CRK4-depleted parasites, β-tubulin was not differentially phosphorylated (22). A potential other mechanism involves proteins of the kinesin 13 family. These conserved motor proteins possess microtubule depolymerizing activity and function during mitosis in other organisms (40, 41). *P. falciparum* kinesin 13 was less phosphorylated in *Pf*CRK4-depleted parasites (22), but a potential role of kinesin 13 for microtubule rearrangement remains unclear.

Profiling the *Pf*CRK4 proximity proteome identified 44 proteins in close proximity to the kinase, including *Pf*SPB, the first described protein associated with the intranuclear part of the centriolar plaque (Fig. S2E). Three phosphorylations of *Pf*SPB (S68, S1967, and S2195) depend on *Pf*CRK4 (22), but their functional significance remains to be tested. In addition, this set of candidates also informed on how *Pf*CRK4 kinase activity may be regulated. Based on the presence of a conserved amino acid motif in *Pf*CRK4, the pleiotropic kinase *Pf*CK2 was previously predicted to phosphorylate *Pf*CRK4 at serine 977 (42). *Pf*CK2 is essential for parasite proliferation in the blood stage (43) and we found the catalytic alpha subunit of *Pf*CK2 (PF3D7_1108400) in the proximity of *Pf*CRK4 (Fig. S2B; Table S1A), supporting a role for *Pf*CK2 in regulating the kinase activity of *Pf*CRK4. We also found the putative CDK-regulatory subunit (PF3D7_0105800) enriched in the vicinity of *Pf*CRK4 (Fig. S2B; Table S1A), but its potential role remains elusive. Besides transacting factors, autophosphorylation likely also plays a role for the regulation of kinase activity, as *Pf*CRK4 itself is less phosphorylated in *Pf*CRK4-depleted parasites (22).

The asynchrony during nuclear multiplication suggests that the kinase activity of *Pf*CRK4 is regulated at the level of individual nuclei, which is supported by the uneven distribution of *Pf*CRK4 among nuclei as well as inside nuclei (Fig. 2; Fig. S1D and S4). In analogy to canonical cell cycle kinases (18, 44, 45), *Pf*CRK4 kinase activity is likely altered during parts of the nuclear cycle to allow for genome segregation and licensing of origins.

More studies are needed to elucidate how the kinase activity of *Pf*CRK4 is regulated during asynchronous nuclear cycles and to further analyze its role for other stages of the life cycle (22). Depletion of *Pf*CRK4 had no detectable effect on the development of male gametes, which includes DNA replication, but subsequent parasite development in the mosquito was impaired (22). Recent work has shown that DNA replication and possibly nuclear division during male gamete development are controlled by the related kinase CRK5 (46). Interestingly, CRK5 appears not essential for proliferation in erythrocytes (46, 47), but if and how both kinases interact is unknown.

Key transitions of the *P. falciparum* nuclear cycle, such as entry into S-phase, are likely controlled (4). In the absence of canonical cell-cycle checkpoints in *P. falciparum* (15), linking entry into S-phase and the initiation of mitotic events via *Pf*CRK4 may be an alternative means to exert control over the nuclear cycle and, in turn, parasite proliferation. In addition, this work renders *Pf*CRK4 a major regulator of nuclear-cycle progression and further support its significance as target for chemotherapeutic intervention to curb malaria.

## MATERIALS AND METHODS

### *P. falciparum* cell culture

*P. falciparum* 3D7 parasites were grown in fresh O, Rh+ erythrocytes at 4% hematocrit in RPMI 1640 medium supplemented with 0.5% AlbuMAX II (Gibco by Life Technologies, Paisley, UK), 0.2 mM hypoxanthine (CCPro, Oberdorla, Germany), 25 mM HEPES (Merck KGaA, Darmstadt, Germany) pH 7.4, and 12.5 µg/mL gentamicin (Carl Roth, Karlsruhe, Germany), at 37°C in 90% relative humidity, 5% O_2_, and 3% CO_2_ (48). Routine synchronizations were performed by 5% sorbitol treatment as previously described (49). For tight synchronization (1–3 h), a combination of sorbitol and heparin (50 IU/mL) was used (50). Briefly, trophozoite cultures were grown in the presence of 50 IU heparin (Merck KGaA, Darmstadt, Germany) until most parasites were at the late schizont stage. Heparin was then washed out and parasites were allowed to reinvade into fresh erythrocytes for 1–3 h with gentle agitation at 60 rpm. After the invasion period, a sorbitol treatment was performed to remove unruptured schizonts. Parasite genomic DNA extraction was performed on 100–200 µL washed erythrocyte pellet with a parasitemia of 1–5%, using the DNeasy blood & tissue kit (Qiagen GmbH, Hilden, Germany).

### Cloning of DNA constructs for parasite transfection

Primers used in this study are listed in Table 1. The C-terminus of *Pf*CRK4 (PF3D7_0317200) was tagged with BirA* or GFP by single crossover homologous recombination using the selection-linked integration (SLI) strategy (51). For the *Pf*CRK4::BirA* construct, we first amplified the BirA* sequence from MDV1/PEG3::BirA* (52), using primers P1/P2 and cloned into a pSLI-TGD-based plasmid (a kind gift from Tobias Spielmann) using Mlu1 and SalI restriction sites. Subsequently, the C-terminal 800 bp of *Pf*CRK4 (without stop codon) was PCR amplified from *P. falciparum* 3D7 gDNA using primers P3/P4 and ligated into the NotI/MluI digested pSLI-TGD::BirA* plasmid, giving rise to the pSLI-*Pf*CRK4::BirA* plasmid. For the *Pf*CRK4::GFP plasmid, the same 800 bp homology region of *Pf*CRK4 (P3/P5) was cloned into pSLI-GFP-glmS (derived from pSLI-TGD) using the NotI/MluI digested backbone and Gibson assembled.

**TABLE 1:** Primer used in this study

Generation of *Pf*CRK4::BirA* parasite line	
P1	GCACGCGTAAAGATAATACAGTACCATTAAAATTAATAGC
P2	ACGCGTCGACCAGATCCTCTTCTGAGATGAGTTTTTGTTCAGATCC
P3	ATCAGCACTCACAGTTGGGTAAGACTTTCCCGTACGACGCGTAAAGTAATATGTTCCGTTATCTTTGTAATATCTCAACAAATCTTCATTCAAATGTAGG
P4	CCAAGCTATTTAGGTGACACTATAGAATACTCGCGGCCGCTGAAAGGATTGATATGACATATGACCATATACGTAATTATGTAAAATATGTTTATTTACC
P5	TGCACCTCCAGCACCAGCAGCAGCACCTCTAGCACGCGTAAAGTAATATGTTCCGTTATCTTTGTAATATCTCAACAAATCTTCATTCAAATGTAGG
P6	CAGCACATAAAGCAATGTACAGC
P7	CTGTAAATACATCTACACCCC
P8	CACACAGGAAACAGCTATGACC
P9	GCCAGTTTGTATGATAGACAGCG
P10	GCATCACCTTCACCCTCTCC

Generation of ***Pf*** SPB::Halo parasite line	
P11	GGGAATTTCCTTATAGGGCCTTTCCTTCAATTTCGATATCAATTTATAGAAAC
P12	GGCGACGCTGTAATCCTCAGAGATGG
P13	AATACGACTCACTATAGGGCGACTCACTATAGAATTCTAGATTTAATAAATATG
P14	CCCATCTCTGAGGATTACAGCGTCG
P15	TATTATTATTTATGTGTAAAGGAGA
P16	AAACTCTCCTTTACACATAAATAAT
P17	GAACATATTTATTAAATCTAGCCAAATGAATACAGGAATAATATAAT
P18	TGCATTATATTTACTACTGTATCTAC
P19	ACAGTAGTAAATATAATGCACCTAGGCCAACCACTG
P20	TATATGTGTGATGAACCTTTTTAACCGGAAATCTCCAGAGTA
P21	AAAGGTTCATCACACATATATT
P22	CGAAAAGTGCCACCTGACGTCTTAACATTCGTATGGTGTTCA
P23	TCATGATACACCATCTCCACTGCATATAAATAATTTTAATCAAGATG
P24	CATCTTGATTAAAATTATTTATATGCAGTGGAGATGGTGTATCATGA
P25	TCACATGCAAACAAGCAATAATC
P26	GTTATCAACTTGGCACGCC

Diagnostic PCR was performed to test for integration of the BirA* (5′ integration: P6/P7, 3′ integration: P8/P9, and WT locus: P6/P9) and GFP (5′ integration: P6/P10, 3′ integration: P8/P9, and WT locus: P6/P9) targeting constructs. A schematic representation of endogenous *Pf*CRK4, the constructs, and the recombined locus is shown in Fig. S1.

Endogenous tagging of *Pf*SPB (PF3D7_0303500) with a Halo tag was performed by CRISPR/Cas9-mediated homologous recombination using the pDC2-cam-coCas9-U6.2-Hdhfr plasmid (kind gift from Marcus Lee) following previously described methodology (53, 54) and schematically shown in Fig. S2C. To tag *Pf*SPB in the *Pf*CRK4::GFP background, which expresses the hDHFR cassette, we first modified the pDC2-cam-coCas9-U6-hDHFR plasmid by replacing the hDHFR resistance marker for a blasticidin (BSD) resistance cassette, producing the construct pDC2-cam-coCas9-U6-BSD. The sequence of the BSD resistance cassette was amplified from the vector p3xNLS-mCherry (12).

To remove the BbsI cut site within the BSD gene, which would interfere with the insertion of the gRNAs, we introduced a silent mutation to destroy the BbsI restriction site. For this, the BSD cassette was amplified as two fragments overlapping at the restriction site (P11/P12 and P13/P14), with the mutation introduced in the respective overlapping forward and reverse primers and cloned into the EcoRI-ApaI sites of the vector, ultimately creating the vector pDC2-cam-coCas9-U6.2-BSD. The selection of gRNA sequences, designed using the Eukaryotic Pathogen CRISPR guide RNA/DNA Design Tool (55), was based on the distance to the C-terminus of *Pf*SPB and the predicted activity scores. gRNAs carrying overhangs complementary to the BbsI cut site (P15/P16) were annealed and phosphorylated before ligation into the BbsI digested pDC2-cam-coCas9-U6.2-BSD plasmid.

Next, the left and right homologous arms (donor template) were amplified separately by PCR from genomic DNA of *P. falciparum* 3D7. In total, four overlapping fragments were amplified: the 5′ homology region as two parts to include protective silent mutations, which avoid re-cutting after recombination (P17/18 and P23/P24), the 3′ homology region (P21/P22), as well as the Halo tag sequence with a stop codon (P19/P20). These fragments were Gibson assembled and ligated into the EcoRI and AatII digested plasmid (Fig. S2C).

Primers were purchased from Thermo Fisher Scientific or Merck. Restriction enzymes were purchased from NEB. All PCRs were performed using Phusion DNA polymerase (New England Biolabs GmbH, Frankfurt, Germany). Molecular cloning was performed using HiFi DNA Assembly (New England Biolabs GmbH, Frankfurt, Germany), T4 DNA Ligase (New England Biolabs GmbH, Frankfurt, Germany), or Gibson Assembly (New England Biolabs GmbH, Frankfurt, Germany). Correct sequence of inserts was verified by Sanger sequencing.

### Transfection of *P. falciparum*

For transfection, young ring-stage *P. falciparum* 3D7 parasites at 5–8% parasitemia were transfected with 75–100 µg of purified plasmid DNA as previously described (56). Electroporation was performed with a Gene Pulser Xcell, Bio-Rad (settings: 0.31 kV, 0.950 µF, capacitance set to “High Cap,” resistance on the Pulse Controller II set to “Infinite”). To select for parasites carrying the genomic modification via the SLI system, we followed the previously published protocol (51). In brief, a first selection for episomally maintained plasmids was carried out using 2.5 nM of WR99210 (kind gift of Jacobus Pharmaceutical Company, Princeton, USA), followed by treatment with 800 µg/mL geneticin (Thermo Fisher Scientific Inc., Hercules, USA) for genome integration selection. Limiting dilution was done to obtain clonal parasite lines. For the CRISPR/Cas9 approach, selection was performed with 5 µg/mL blasticidin S (InVivoGen, San Diego, USA). Unless otherwise noted, *Pf*CRK4::HA::DD parasites were cultured in the presence of 250 nM Shield-1 (22).

### Live- and fixed-cell imaging

#### Immunofluorescence assay

Immunofluorescence staining for confocal microscopy of blood-stage parasites was done as previously described with minor alterations (12). In brief, parasitized erythrocytes were seeded on poly-L-lysine coated ibidi eight-well chambered glass bottom dish and fixed with 4% paraformaldehyde/phosphate-buffered saline (PFA/PBS) at 37°C for 20 min. Cells were then permeabilized with 0.1% Triton X-100/PBS for 15 min at room temperature, rinsed three times with PBS and incubated with 0.1 mg/mL NaBH_4_/PBS solution for 10 min to quench free aldehyde groups. After washing three times with PBS, cells were blocked with 3% bovine serum albumin (BSA)/PBS for 30–60 min and incubated with primary antibodies diluted in 3% BSA/PBS for 2 h at room temperature or overnight at 4°C. Next, the cells were washed three times with 0.5% Tween-20/PBS and incubated with secondary antibodies plus Hoechst in 3% BSA/PBS for 1 h at room temperature. After washing twice with 0.5% Tween-20/PBS and once with PBS, the cells were directly imaged or stored in PBS at 4°C in the dark until imaging.

For the *Pf*SPB::Halo parasite line, labeling was performed in live cells according to the manufacturer’s instructions (Promega, Walldorf, Germany). In brief, parasites were labeled for 1 h at 37°C in standard culture conditions with 5 µM of the cell-permeable HaloTag TMR ligand. After labeling, the cells were rinsed with fresh prewarmed imaging media for 30 min, fixed, and processed for immunofluorescent staining as described above.

Image acquisition was done using a Leica TCS Sp8 scanning confocal microscope using the HC PL APO CS2 63 ×/1.4 N.A. oil immersion objective. Unless otherwise stated, images were captured as a series of z-stacks separated by 0.2–0.4 μm intervals and representative confocal images are shown as maximum projections. Images were processed with the lightning algorithm in the adaptive mode (except for Fig. 1 and 2; Fig. S1) using default settings with further analysis using Fiji (57). Brightness and contrast were adjusted for visualization.

### Analysis of the cellular distribution of *Pf*CRK4

To quantify the *Pf*CRK4 distribution relative to microtubules, the signal density of *Pf*CRK4::GFP was determined in different cellular compartments and at different microtubule structures as previously described (Fig. 2; Fig. S4 and S5) (3, 58, 59). In brief, areas were defined by the presence of different markers. Specifically, the nuclear compartment was defined as the area with a high Hoechst 33342 signal and microtubules as the areas with a high α-tubulin antibody signal. The whole parasite (including the nucleus but without the food vacuole) was defined as the area where there was weak, unspecific background signal of the α-tubulin antibody, which was only present in the parasite but not the red blood cell, seen by comparison with the brightfield channel. The cytoplasm compartment was set as the whole parasite (as defined above) without the areas that showed a high Hoechst 33342 signal, that is, the nuclei.

The different areas were isolated by creation of specific masks from the reference channels through thresholding. Before thresholding, a Gaussian blur with a radius of 2 pixels was applied to the reference channels. Subsequently, the reference channels were thresholded in the default mode with the following parameters: nuclear mask (Hoechst 33342 channel), threshold of 30/255; parasite mask (α-tubulin channel), threshold of 10/255; and microtubule mask (α-tubulin channel), threshold of 50/255. For the cytoplasm mask (parasite mask minus the nucleus mask), the area of the nucleus mask was removed from the corresponding parasite mask by inverting the nucleus mask and multiplying it with the cytoplasm mask via the image calculator function of Fiji.

The reference masks were then individually applied to the unmodified *Pf*CRK4 channel and all *Pf*CRK4 signal outside of the respective mask was deleted. The remaining *Pf*CRK4 signal in the different z-slices was added using the Sum Slices mode of the z-projection function in Fiji and measured as the raw integrated density. To obtain the signal density within the different compartments, the raw integrated density of *Pf*CRK4 within that compartment was divided by the area of the respective mask that was applied. The area of the respective masks was determined by thresholding the binary masks, measuring the area of the threshold and summing the area of the different z-slices. *Pf*CRK4 signal densities within the different compartments were normalized to the signal density within the parasite area.

To stratify the different microtubule structures, the microtubule masks were manually segmented in 3D by removing all areas that did not belong to either an elongated or a focal microtubule signal. Subsequently, these masks containing either those areas of elongated or focal microtubules were used to isolate the total signal of *Pf*CRK4 in only the selected microtubule structures and the signal within these masks was measured as detailed above. For comparison of *Pf*CRK4 signal density at different microtubule structures within the same parasite, *Pf*CRK4 signal densities at different microtubule structures were normalized to the mean signal intensity of *Pf*CRK4 at all microtubule structures within the same cell.

### Live-cell imaging

For live-cell imaging, we followed previously published protocols with minor modifications (3). In brief, sterile glass bottom eight well-dishes (Ibidi GmbH, Martinsried, Germany) were coated with 5 mg/mL Concanavalin A (Merck KGaA, Darmstadt, Germany) and rinsed with PBS. About 500 µL of resuspended parasite culture was washed twice with incomplete RPMI and left to settle on the dish for 10 min at 37°C before unattached cells were washed off using incomplete RPMI until a monolayer remained. Cells were left to recover at standard culturing conditions in complete RPMI for at least 8 h before media were exchanged to the phenol red-free complete RPMI imaging medium [RPMI 1640 L-Glutamine (PAN-Biotech, Aidenbach, Germany), 0.5% AlbuMAX II, 0.2 mM Hypoxanthine, 25 mM HEPES pH 7.3, and 12.5 µg/mL gentamicin] that had been equilibrated to incubator gas conditions for at least 6 h.

Cells were incubated with SPY555 or SPY650-tubulin (Spirochrome, Stein am Rhein, Switzerland) in phenol red-free complete RPMI imaging medium starting from 2 h before the start of imaging. For experiments presented in Fig. 3 and movies S1 and S2, the cells were additionally incubated with 5-SiR-Hoechst kindly provided by Gražvydas Lukinavičius and Jonas Bucevičius (60). For *Pf*CRK4 rescue experiments, *Pf*CRK4::HA::DD was stabilized at the same time as the addition of the live-cell compatible dyes by the addition of 250 nM of Shield-1 within the imaging medium. Dishes were completely filled with imaging media, closed, and sealed tightly using parafilm before imaging started.

Point laser scanning confocal microscopy was performed on a Zeiss LSM900 microscope equipped with an Airyscan 2 detector using Plan-Apochromat 63×/1.4 oil immersion objective. Live-cell imaging was performed at 37°C. Images were acquired at multiple positions using an automated stage and the Definite Focus module for focus stabilization with a time-resolution of 15 min/stack for 2–5 h for correlation of microtubule structure with DNA content (Fig. 3; movies S1 and S2), a time-resolution of 10 min/stack for 15 h for correlation of microtubule structures and DNA replication (Fig. 4A through C; movie S3) and a time resolution of 20 min/stack for 10 h for analysis of microtubule structures upon aphidicolin (Fig. 4F and G). Multichannel images were acquired sequentially in the line scanning mode using 561 and 640 nm diode lasers for SPY555-tubulin and 5-SiR-Hoechst/SPY650-tubulin imaging, respectively. Emission detection was configured using variable dichroic mirrors to be 490–650 for SPY555-tubulin and 490–700 for 5-SiR-Hoechst/SPY650-tubulin detection. The Airyscan detector was used with the gain adjusted between 700 and 900 V, offset was not adjusted (0%). Brightfield images were obtained from a transmitted light PMT detector, with the gain adjusted between 700 and 900 V. Sampling was Nyquist-optimized in xy-axis (approx. 50 nm) and 500 µm in z-axis, bidirectionally with pixel dwell time between 0.7 and 1.2 μs and 2× line averaging. Subsequently, ZEN Blue 3.1 software was used for 3D Airyscan processing with automatically determined default Airyscan Filtering strength.

### Analysis of live-cell imaging to correlate microtubule structures with DNA content

Processing and analysis of imaging files were done with Fiji (57), analysis and plotting of data in Microsoft Excel and GraphPad Prism 8, respectively. Multiposition images were manually inspected for dead or abnormal parasites using the brightfield channel. Individual parasites were saved as 300 × 300 pixels TIFF files containing original channel, time, and z-slice information. Parasites were further evaluated for nuclei number and occurrence of hemispindle structures. Only parasites containing one nucleus and distinct hemispindle structures were chosen for further analysis.

Time-lapse images were stabilized in xy using the Register Virtual Stack Slices Plugin in Fiji. Briefly, for each parasite, a time-lapsed reference z-slice of the brightfield channel was registered via the translation mode (no deformation) at standard parameters. The resulting transformation matrixes were saved. The transformation matrixes were then applied to all other z-slices in all other channels using the Transform Virtual Stack Slices Plugin in Fiji.

For analysis of DNA content and microtubule structures (Fig. 3C through F), a tight selection was first applied around the parasite to remove unspecific background signal outside of the infected red blood cell, which was particularly strong in the 5-SiR-Hoechst channel. For this, an oval mask of varying size was applied around the parasite in the 5-SiR-Hoechst channel and all signals outside of the mask were deleted. To further subtract background and segment the nucleus, a second mask was applied, which was created from the background-subtracted 5-SiR-Hoechst channel by applying a median filter with a radius of 2 pixels and a threshold of 550 or 300 depending on signal intensity. The resulting mask was applied to the 5-SiR-Hoechst channel, all signal outside the mask deleted, the signal in the different z-slices of the resulting image was summed using the z-projection Sum Slices function and the remaining raw integrated density was measured. For analysis of the DNA content over time, the raw integrated density was normalized to the first time point of the timelapse of the individual parasites.

For visualization of the different microtubule structures, a collage of the SPY555-tubulin channel over time was done. A field of 100 × 100 pixels centered around the spindle structures in a maximum intensity z-projection of the SPY555-tubulin channel was cut out and the resulting images were combined in a single row via the make montage function in Fiji.

All antibodies and dyes used in this study are detailed in Table 2.

**TABLE 2:** Antibodies and dyes in this study

Antibody	Species	Dilution	Source
Anti-alpha-tubulin B-5-1-2, monoclonal (T5168)	Mouse	1:500	Merck KGaA, Darmstadt, Germany
Anti-PfCentrin3, polyclonal	Rabbit	1:500	Ref. 6
Anti-mouse Alexa Fluor Plus 488	Goat	1:500	Thermo Fisher Scientific Inc., Waltham, USA
Anti-mouse-Atto647	Goat	1:1,000	Merck KGaA, Darmstadt, Germany
Anti-rabbit Alexa Fluor Plus 488	Goat	1:1,000	Thermo Fisher Scientific Inc., Waltham, USA
Anti-rabbit-Atto647	Goat	1:1,000	Merck KGaA, Darmstadt, Germany
aAnti-rabbit Alexa Fluor Plus 488	Goat	1:1,000	Thermo Fisher Scientific Inc., Waltham, USA
Anti-rabbit-GFP, monoclonal (G10632)	Recombinant	1:40	Thermo Fisher Scientific Inc., Waltham, USA
Streptavidin Alexa Fluor 647	–	1:40	Thermo Fisher Scientific Inc., Waltham, USA
Anti-Histone H3 nuclear marker and ChIP grade	Rabbit	1:2,000	Abcam, Amsterdam, Netherlands
Streptavidin—IRDye 800CW	–	1:1,500	LI-COR, Lincoln, USA
Anti-rabbit IRDye 680CW	Goat	1:10,000	LI-COR; Lincoln, USA
**Dye**	**Dilution/concentration**		**Source**
SPY555-Tubulin (SC203) SPY650-Tubulin (SC503)	1:2,000		Spirochrome, Stein am Rhein, Switzerland
5-SiR-Hoechst	22 nM		Ref. 48
Hoechst33342	1:1,000		Thermo Fisher Scientific Inc., Waltham, USA
HaloTag TMR Ligand	5 µM		Promega, Walldorf, Germany

### Cell culture and harvest of biotinylated proteins

Experiments were performed as described previously with minor modifications . To label biotinylated proteins in parasites for analysis by fixed imaging, streptavidin blot, or mass spectrometry, sorbitol synchronized ring-stage *Pf*CRK4::BirA* and 3D7 WT parasites were cultured [+] or [−] of 50 µM biotin (Merck KGaA, Darmstadt, Germany) supplementation. Cultures were harvested for analysis 24 h later as the majority of parasites had multiple nuclei.

For mass spectrometry identification of biotinylated proteins, ~1×10^9^ parasites (4 mL of packed red blood cells with 8–10% parasitemia) were isolated in triplicate for each condition. Uninfected erythrocytes were removed using saponin buffer [0.1% saponin/PBS plus 1× cOmplete protease inhibitor cocktail (Roche, Basel, Switzerland)]. Cells were washed twice with saponin buffer to further remove erythrocytes and erythrocyte debris, followed by PBS plus 1× cOmplete protease inhibitor cocktail and 1 mM phenylmethylsulfonyl fluoride. Parasite pellets were resuspended in 1 mL of RIPA lysis buffer [50 mM Tris, 150 mM NaCl, 0.1% SDS, 0.5% sodium deoxychlorate, 1% NP-40, 1 mM EDTA, 1 mM dithiothreitol (DTT), and 1× cOmplete protease inhibitor] for 15 min and sonicated in a water bath Bioruptor Plus (10 cycles of 30 s ON and 30 s OFF pulses at high intensity). The lysates were further clarified by centrifuging at 15,000× *g* for 15 min at 4°C to remove hemozoin and other insoluble debris. About 100 µL of streptavidin magnetic beads (MagResyn Streptavidin beads) was washed twice with 0.1% BSA in PBS and blocked with 2.5% BSA in PBS for 1 h at room temperature with gentle agitation on a rotating wheel. Beads were washed twice with RIPA buffer and incubated with 1 mL of each sample lysate overnight at 4°C with gentile agitation. After incubation, beads were sequentially washed thrice with RIPA buffer, thrice with SDS buffer (2% SDS, 50 mM Tris-HCl, pH 7.4, and 150 mM NaCl), thrice with Urea buffer (8 M Urea, 50 mM Tris-HCl, pH 7.4, and 150 mM NaCl) and resuspended in 400 µL of 100 mM ammonium bicarbonate buffer, pH 8.0, and stored at −80°C until further processing. A fraction of the beads samples were taken for Western blot analysis.

### SDS page and Western blot analysis

Samples for Western blot analysis were supplemented with 4× Laemmli buffer (Bio-Rad Laboratories, Hercules, USA) and 2.5% β-mercaptoethanol. After incubation for 10 min at 95°C, beads were removed via a magnetic rack and proteins were loaded on a 4–15% Mini-PROTEAN TGX Precast Protein Gel (Bio-Rad). Blotting was performed using the Trans-Blot Turbo Mini 0.2 µM Nitrocellulose Transfer Pack (Bio-Rad) on a Trans-Blot Turbo Transfer System (Bio-Rad). The membrane was blocked with 3% BSA in TBST (0.05% Tween-20/PBS) for 1 h and then probed with α-histone H3 antibody in 1% BSA/TBST for 1 h at room temperature. After three washes with TBST for 10 min, the membrane was stained with the secondary antibodies IRDye 800CW-labeled streptavidin and IRDye 680CW goat anti-rabbit IgG in 1% BSA/TBST supplemented with 0.05% SDS for 1 h at room temperature (Table 2). After three 10-min washes with TBST and one 5-min wash with PBS, fluorescent signals were visualized using an LI-COR Odyssey CLx Imaging System.

### Label-free quantitative mass spectrometric analysis

Label-free quantitative mass spectrometry and data analysis were performed as commercially available service by the Proteome Factory, Berlin, Germany (www.proteome-factory.com) according to standard procedures.

### On-beads trypsin digestion

Proteins bound to magnetic beads were cleaved by trypsin according to standard procedures. In brief, magnetic beads were washed two times with 45 µL ABC buffer (ammonium bicarbonate buffer, 50 mM, and pH 8.0), then resuspended in further 45 µL ABC. Reduction of cysteine residues was performed by addition of 5 µL 100 mM DTT and incubation for 1 h at 60°C. For alkylation, 6 µL 100 mM 2-iodoacetamide was added and incubated for 1 h at room temperature. Residual alkylating agent was quenched by further addition of 100 mM DTT. About 500 ng of trypsin was added, and proteolysis was allowed to proceed overnight at 37°C. The solution was acidified by addition of formic acid and the supernatant was collected. Samples were stored at −80°C until analysis.

### LC-MS/MS analysis

Label-free quantitative proteomics using LCMS/MS was performed using an Ultimate 3000 nanoHPLC system. Samples were loaded onto a trapping column (kept at 35°C) for desalting (Thermo Fisher Scientific, Dionex, Pepmap C18) and peptides separated on a 500 × 0.075 mm^2^ column (0.5 µL/min, 50°C, Reprosil C18-AQ, Dr. Maisch) using a linear gradient from 10% to 32% B (solvent A: water, solvent B: acetonitrile, both with 0.1% formic acid). The column effluent was directed to an Orbitrap Velos mass spectrometer via a nanoelectrospray ion source. Survey scans were detected at a nominal resolution of *R*=60,000, and up to 10 MS/MS spectra from ions of interest (charge states +2 and above) were data-dependently recorded. Total acquisition time was 300 min per analysis.

### Database search and analysis

RAW data were processed with the MaxQuant software package (version 1.6.14.0). MSMS spectra were searched against *P. falciparum* 3D7 and human sequences from RefSeq databases. Cysteine carbamidomethylation was set as a fixed modification, while methionine oxidation and glutamine (Gln) and asparagine (Asn) deamidation were used as variable modifications for database search. Label-free quantification was done with oxidation (M), acetylation (Protein N-term), and deamidation (N) included using the match between runs feature. Default settings were used for further parameters.

Statistical analysis of the proteingroups.txt file from the MaxQuant output was done with Perseus software (version 1.6.15.0). LFQ data were imported, then decoy matches and putative contaminants were removed. Intensity data were log2 transformed, and proteins with at least two valid LFQ values in each group were further analyzed. Imputation using the “imputeLCMD” tool from the PerseusR Package was used with MCARS and MNARS+ where applicable. Student’s *t* test was applied to resulting data and a significance threshold was set to *P* value<0.05. Fold change was calculated from the difference of the mean LFQ values from *Pf*CRK4::BirA* [+] biotin and *Pf*CRK4::BirA* [–] biotin by retransforming log2 values. For negative log2 fold changes, the negative inverse value of the 2^*x*^ value was given.

Applying correction for multiple hypothesis testing using Benjamini-Hochberg or permutation-based FDR resulted in no significantly different protein groups between *Pf*CRK4::BirA* [+] and [–] biotin.

### GO term enrichment analysis

GO term enrichment analysis was performed for molecular function, biological process, and cellular component of both overrepresented and underrepresented proteins using PlasmoDB (www.plasmodb.org, accessed on 2 April 2022). The following parameters were used: evidence: curated; limit to GO slim terms: yes; *P*value from Fisher’s exact test 0.05.

### STRING analysis

Interaction evidence among the overrepresented proteins in the vicinity of *Pf*CRK4 was analyzed using the STRING database, version 11.5 (www.string-db.org, accessed on 2 August 2022) (28). In Fig. S3B, proteins are presented as nodes (circles) connected by lines (Edge), whose thickness represents the strength of the connection based on the STRING database. For simplicity, only direct interactions with *Pf*CRK4 are shown in Fig. S3B. The entire data set is provided in Table S1B. The potential protein–protein interactions were predicted using the interaction sources “experiments,” “neighborhood,” “fusion-fission events,” “co-occurrence and co-expression,” and “data imported from public databases of physical interactions.” A medium confidence cutoff of 0.4 was used.

### Flow cytometry analysis of parasite DNA content and growth rate

Parasites DNA content and growth rate were determined as previously described using SYBR Green I (22, 34, 35). In brief, highly synchronized *Pf*CRK4::HA::DD parasites were grown in the presence or absence of 250 nM of Shield-1 (Fig. S6A) and at 32 hpi, 500 nM of paclitaxel (PTX) or solvent was added to the cultures. For the experiment shown in Fig. 4D and E, *Pf*PCNA1::GFP (3) parasites were incubated with 15 µM of aphidicolin (APH) from 28 hpi onwards. At different time points, 100 µL of resuspended culture was collected and fixed with 4% PFA/PBS and 0.0075% glutaraldehyde for 30 min at room temperature. To reduce RNA-derived background signal, the cells were permeabilized with Triton X-100 (Merck KGaA, Darmstadt, Germany) for 8 min, treated with 0.3 mg/mL Ribonuclease A (Merck KGaA, Darmstadt, Germany) for 30 min and subsequently washed. Cells were stained for 20 min with 1:2000 SYBR green I (Invitrogen by Thermo Fisher Scientific Inc., Waltham, USA) in PBS, washed, and analyzed on a BD FACSCelesta flow cytometer. For all conditions, 500–1,000 infected red blood cells were recorded. To calculate cellular DNA content, fluorescence measurements from single-infected parasites at 24 hpi were defined as 1 *C*. All flow-cytometry data were analyzed with FlowJo and GraphPad software.

## Data Availability

All data needed to evaluate the conclusions in the paper are present in the paper and/or the supplemental material.

## SUPPLEMENTAL MATERIAL

Table S1 mbio.00779-23-s0001.xlsx Proximity-proteomic profiling of *Pf*CRK4.

Video S1 mbio.00779-23-s0002.avi Time-lapse super-resolution microscopy of *Pf*CRK4-depleted parasites showing highly dynamic hemispindle-like structures. Microtubules were labelled with the live-cell compatible dye SPY555-Tubulin (magenta) and DNA with 5-SiR-Hoechst (blue); Transmission image, single slice; fluorescent images, average intensity z-projections; brightness and contrast adjusted for better visibility; time interval, 15 min; scale bar, 5 μM.

Video S2 mbio.00779-23-s0003.avi Time-lapse super-resolution microscopy of parasites with rescued *Pf*CRK4 activity showing rearrangement of microtubules upon restored kinase expression. Imaging conditions as in Video S1; scale bar, 5 μM.

Video S3 mbio.00779-23-s0004.avi Time-lapse super-resolution microscopy of a reporter parasite ectopically expressing nuclear mCherry (blue) and *Pf*PCNA1::GFP (green) and labelled with SPY555-Tubulin (magenta) showing the dynamics of DNA replication and microtubules organization; Transmission image, single slice; fluorescent images, average intensity z-projections; brightness and contrast adjusted for better visibility; time interval, 10 min; scale bar, 5 μM.

Fig S1 mbio.00779-23-s0005.tif Expression of *Pf*CRK4::BirA* leads to biotinylation of proteins largely restricted to the nucleus and its periphery, consistent with the localization of *Pf*CRK4.

Fig S2 mbio.00779-23-s0006.tif Proximity-proteomic profiling of *Pf*CRK4 identified a centriolar plaque protein.

Fig S3 mbio.00779-23-s0007.tif Profiling the proximity proteome of *Pf*CRK4.

Fig S4 mbio.00779-23-s0008.tif Distribution of *Pf*CRK4 relative to nuclear microtubule structures in *P. falciparum* blood stages.

Fig S5 mbio.00779-23-s0009.tif Image analysis workflow to quantify the distribution of *Pf*CRK4 relative to nuclear microtubule structures.

Fig S6 mbio.00779-23-s0010.tif Normal microtubule rearrangement is not a prerequisite for DNA replication.

ASM does not own the copyrights to Supplemental Material that may be linked to, or accessed through, an article. The authors have granted ASM a non-exclusive, world-wide license to publish the Supplemental Material files. Please contact the corresponding author directly for reuse.
