# Supplementary figures and images for: Plasmodium falciparum CRK4 links early mitotic events to the onset of S-phase during schizogony
Source: mBio. 2023 Jun 22;14(4):e00779-23. doi: 10.1128/mbio.00779-23 (PMC10470535; doi:10.1128/mbio.00779-23)

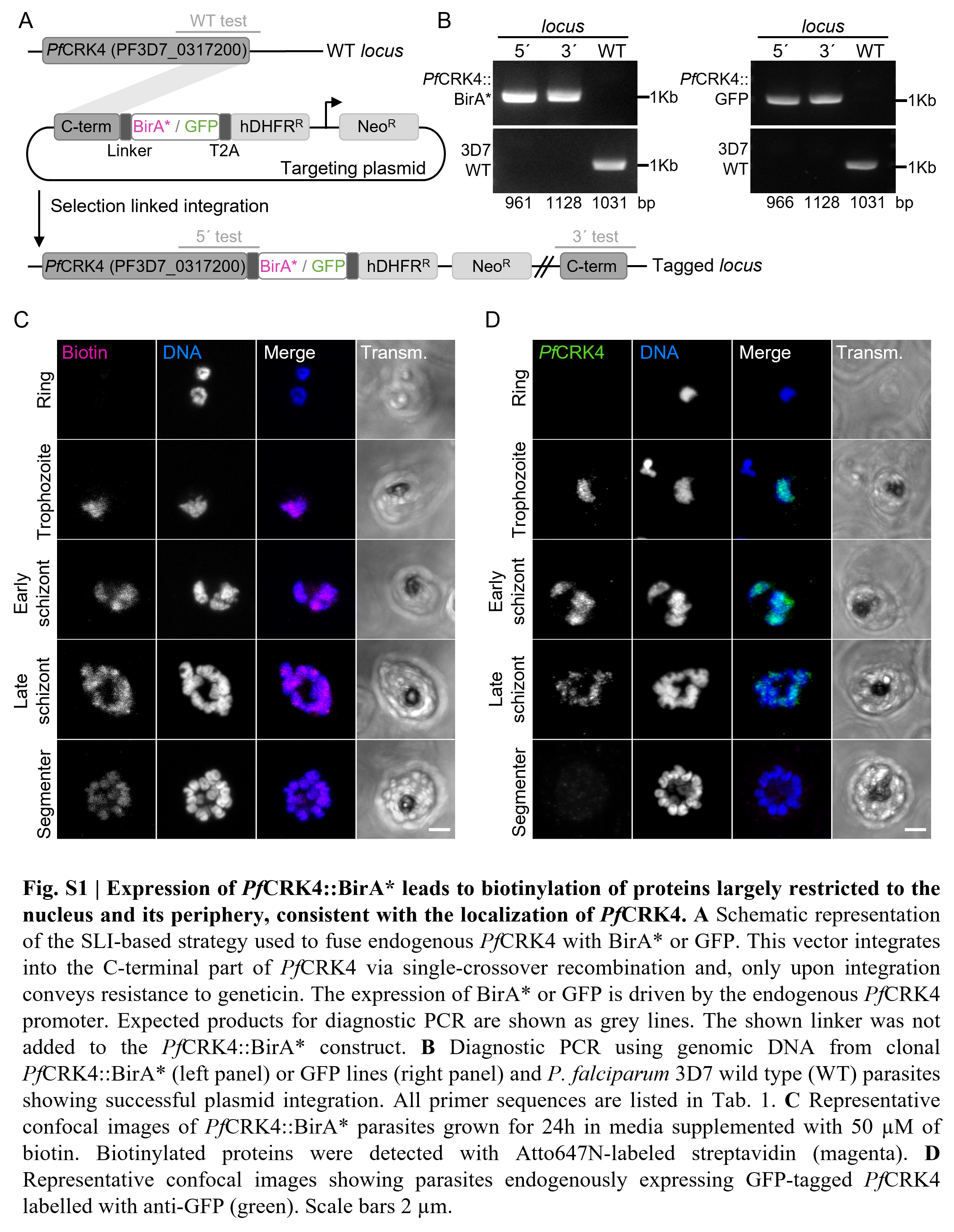

Supplement: Fig S1 — Expression of PfCRK4::BirA* leads to biotinylation of proteins largely restricted to the nucleus and its periphery, consistent with the localization of PfCRK4. [file mbio.00779-23-s0005.tif]

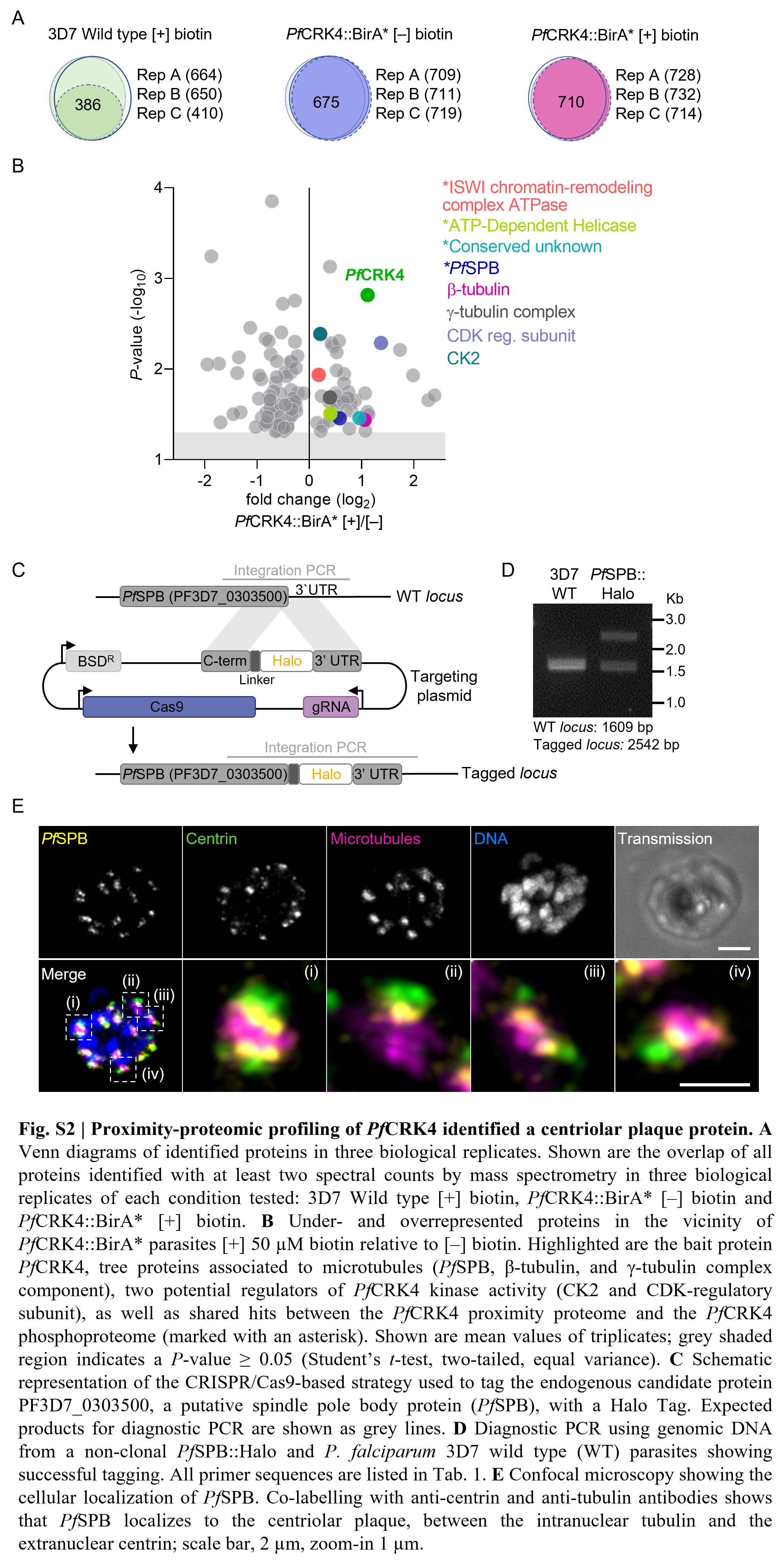

Supplement: Fig S2 — Proximity-proteomic profiling of PfCRK4 identified a centriolar plaque protein. [file mbio.00779-23-s0006.tif]

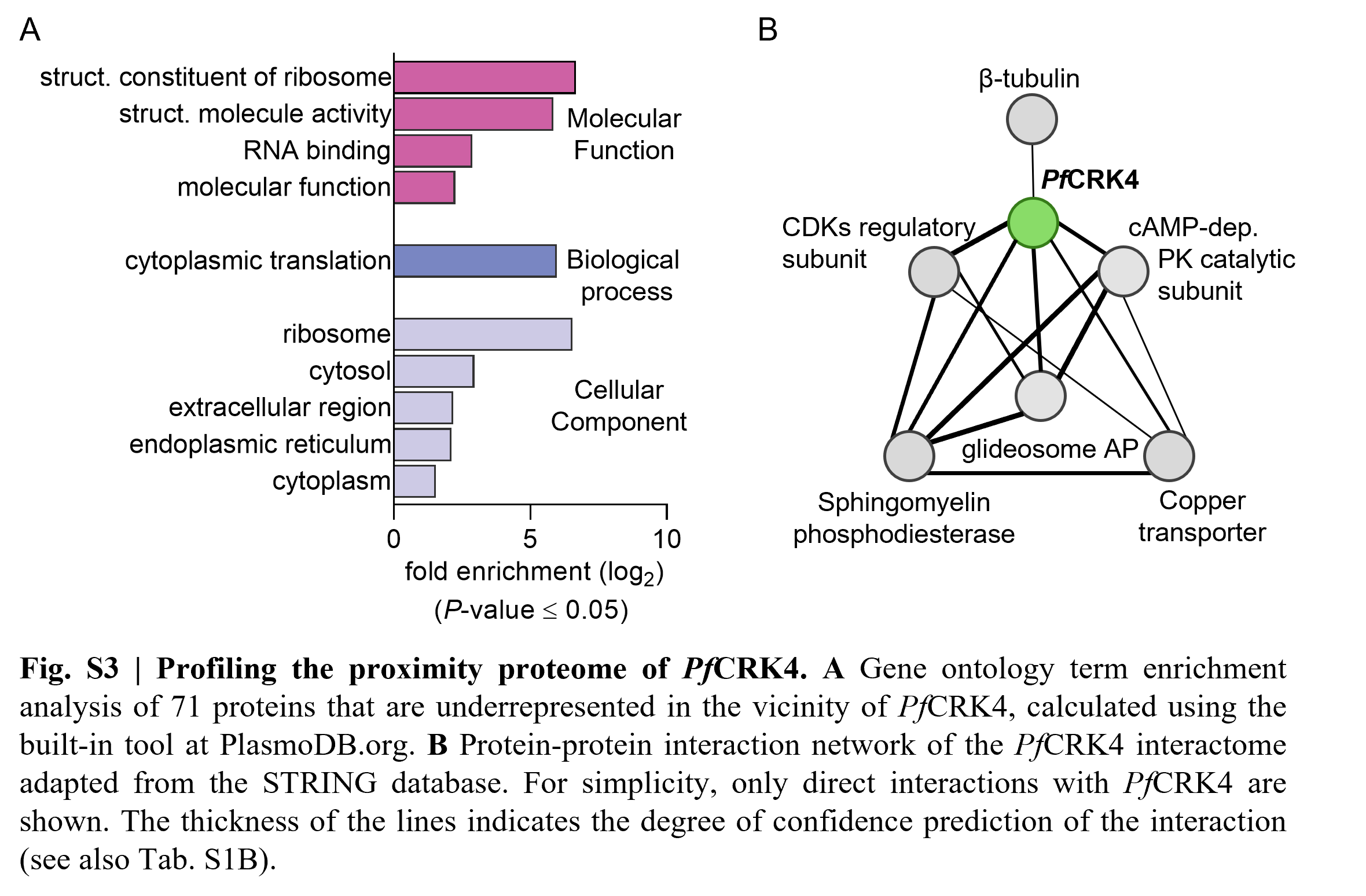

Supplement: Fig S3 — Profiling the proximity proteome of PfCRK4. [file mbio.00779-23-s0007.tif]

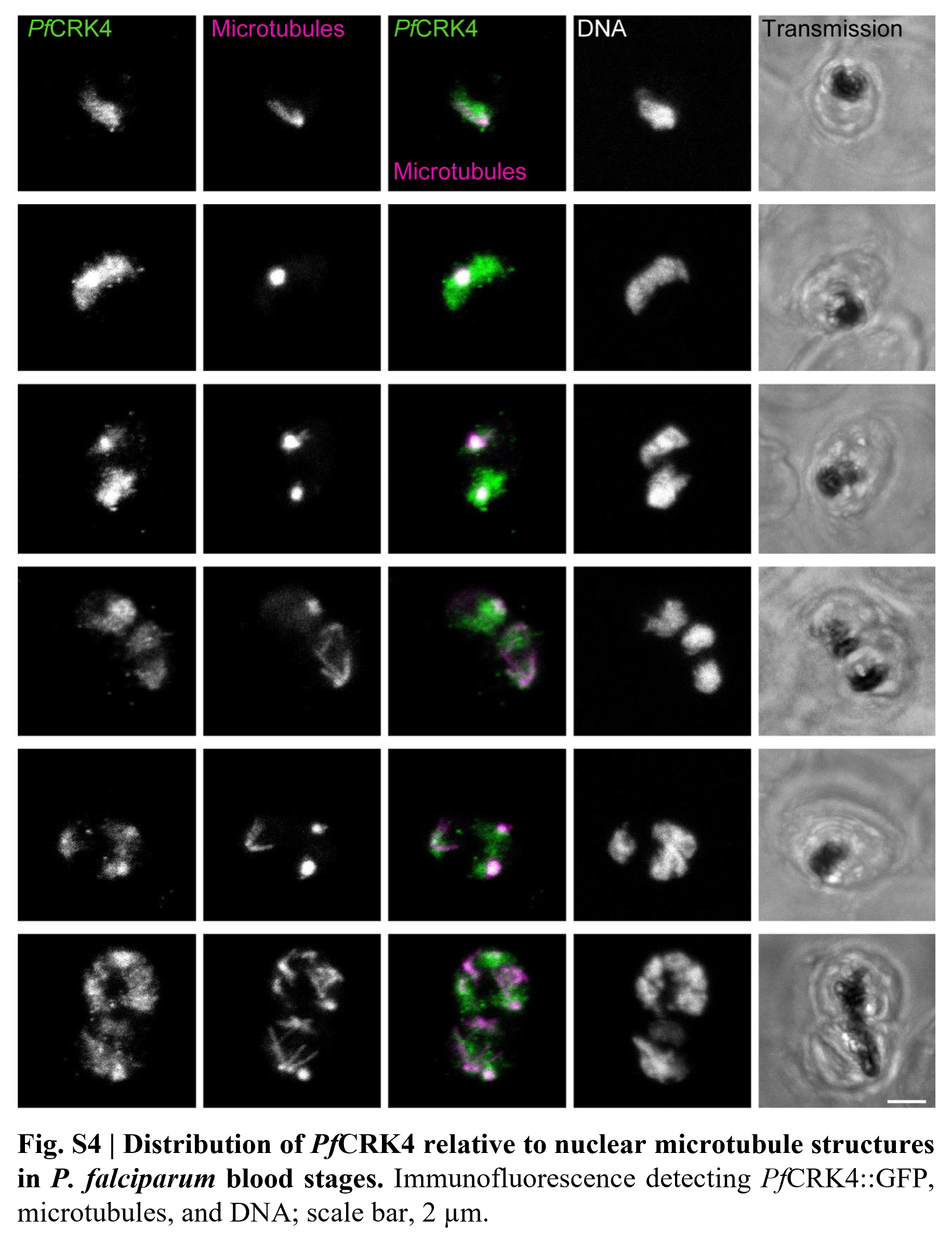

Supplement: Fig S4 — Distribution of PfCRK4 relative to nuclear microtubule structures in P. falciparum blood stages. [file mbio.00779-23-s0008.tif]

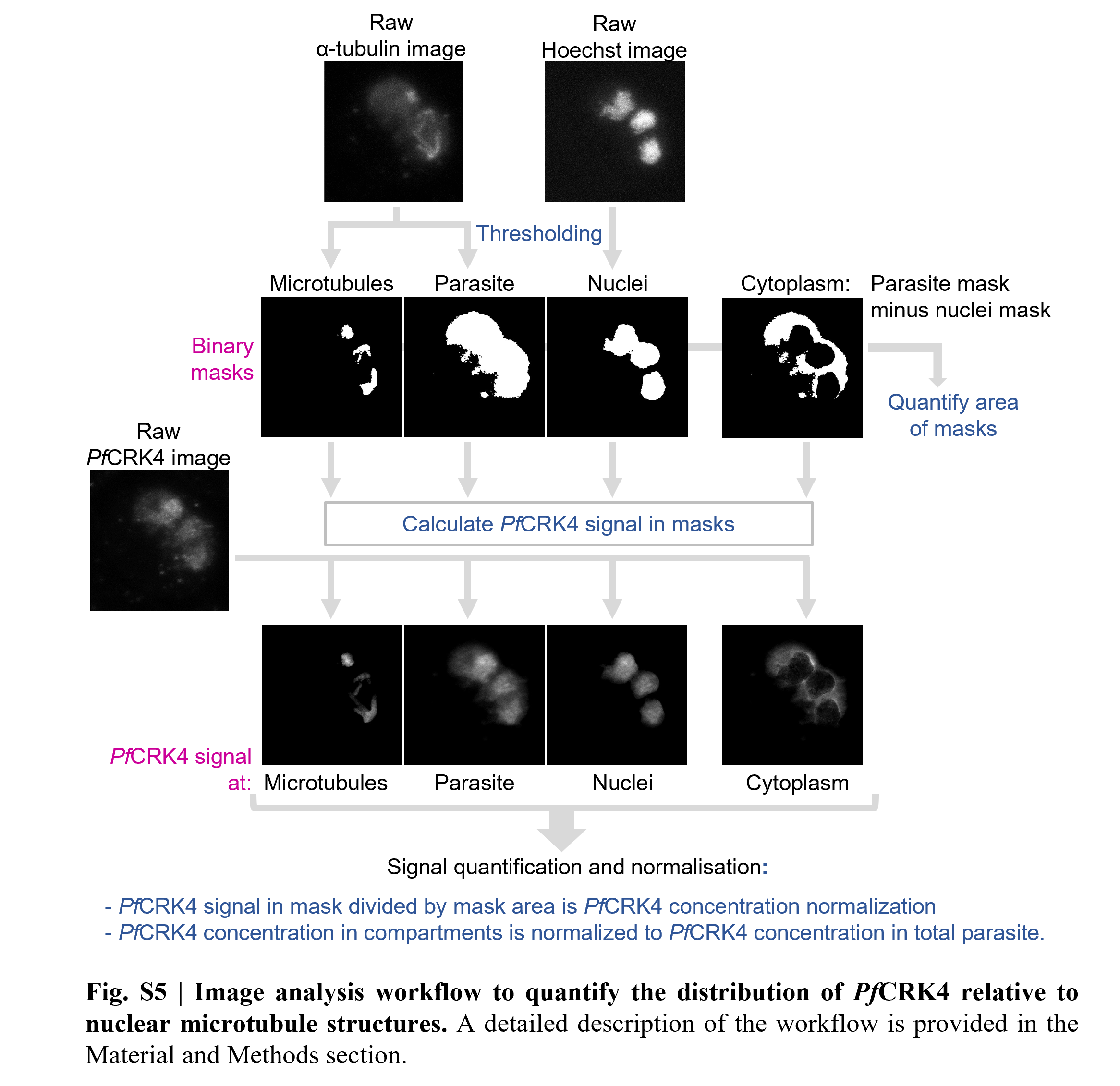

Supplement: Fig S5 — Image analysis workflow to quantify the distribution of PfCRK4 relative to nuclear microtubule structures. [file mbio.00779-23-s0009.tif]

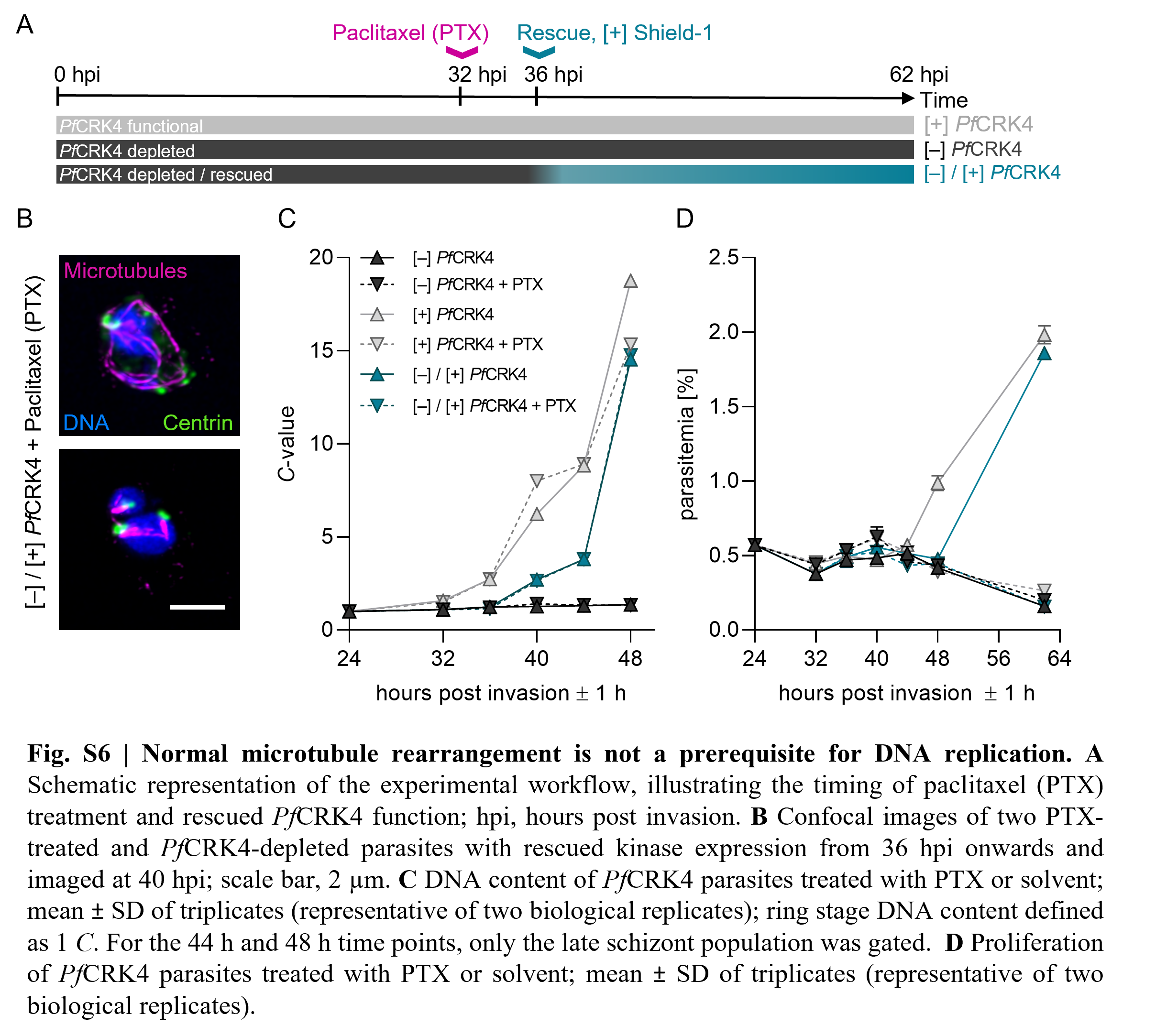

Supplement: Fig S6 — Normal microtubule rearrangement is not a prerequisite for DNA replication. [file mbio.00779-23-s0010.tif]
